# Chromaticity and Optical Characteristics of RF Magnetron-Sputtered Colored Glass for BIPV Applications

**DOI:** 10.3390/nano16140838

**Published:** 2026-07-08

**Authors:** Seungcheol Yoo, Junghyun Kim, Wonseok Choi

**Affiliations:** 1 Department of Electrical Engineering, Hanbat National University, Daejeon 34158, Republic of Korea; aizimhk@naver.com; 2 Department of Advanced Materials Science and Engineering, Hanbat National University, Daejeon 34158, Republic of Korea; jhkim2011@hanbat.ac.kr

**Keywords:** BIPV, color glass, RF magnetron sputtering, ceramic targets, light transmittance

## Abstract

Building-integrated photovoltaics (BIPV) require front-glass materials that satisfy both aesthetic and functional requirements. In this study, colored glass for BIPV applications was fabricated on indium tin oxide (ITO)-coated glass substrates using Radio-Frequency (RF) magnetron sputtering with various ceramic targets, including metal oxides (MoO_3_, WO_3_, and SiO_2_), a nitride (TiN), and Si. The fabricated samples were classified into red, yellow, and blue color families and evaluated in terms of optical transmittance, sheet resistance, and colorimetric properties. The color characteristics were quantitatively analyzed using CIELAB coordinates (L^*^, a^*^, and b^*^) and CIE 1931 chromaticity coordinates (*x*, *y*). The results showed that the chromaticity distributions followed continuous material-dependent trajectories rather than random dispersion. Oxide-based coatings generally shifted toward the yellow region with relatively high lightness, whereas TiN-based coatings shifted toward the blue region with reduced lightness. In addition, transmittance analysis in the photovoltaic-relevant spectral range indicated that Si-based coatings exhibited relatively high optical transparency. The observed color variations are consistent with thin-film interference and the optical properties of the coating materials. This study focuses on the comparative evaluation of optical, electrical, and colorimetric characteristics. This study provides a comparative framework for color realization analysis and offers practical guidance for material selection in colored BIPV glass design.

## 1. Introduction

Over many centuries, buildings have served not only as spaces for human habitation and activity but also as protective envelopes that shield occupants from diverse environmental factors such as temperature, humidity, wind, and solar radiation, thereby ensuring safe and comfortable living conditions. However, in the twenty-first century, the intensification of global climate change and the depletion of energy resources have fundamentally expanded the role of buildings beyond the provision of living space. Buildings are now increasingly expected to contribute to energy conservation, carbon emission reduction, and even the direct production of energy. It is widely recognized within the international community that approximately 40% of global energy consumption occurs in the building sector, and that a substantial share of carbon dioxide emissions also originates from buildings [1,2]. In response to these challenges, governments worldwide have implemented policies that mandate improvements in energy efficiency and the integration of renewable energy technologies within the building sector. The European Union (E.U), for instance, requires newly constructed buildings to achieve nearly zero energy consumption under the Nearly Zero Energy Building (nZEB) regulation. Similarly, Republic of Korea has mandated that all newly constructed buildings with a gross floor area exceeding 500 m^2^ obtain Zero Energy Building (ZEB) certification by 2030 [3]. Within this policy-driven context, building-integrated photovoltaics (BIPV) have emerged as a key technology enabling buildings to generate energy autonomously. Beyond their role in energy saving, BIPV systems have increasingly attracted attention as a multidimensional research topic encompassing building functionality, asset value, and architectural aesthetics [4]. In BIPV systems, colored glass is no longer regarded merely as a decorative element; rather, it is recognized as a critical component that simultaneously influences optical transmittance, color stability, and power generation performance [5].

The core technology of colored BIPV modules lies in the implementation of color on the front glass. To date, existing approaches can be broadly classified into two categories. The first is a printing-based method using ceramic pigments. While this approach offers advantages in terms of process simplicity and low manufacturing cost, it inherently leads to increased reflectance in order to produce color, resulting in reduced optical transmittance and a corresponding decrease in power generation efficiency. In addition, prolonged exposure to solar radiation may cause color fading or performance degradation [6]. The second approach is a coating-based method that exploits thin-film interference effects. In this method, thin films such as metal oxides or nitrides are deposited in thicknesses ranging from several tens to several hundreds of nanometers, selectively reflecting or transmitting specific wavelengths to generate the desired color. This technique has gained increasing attention in recent years due to its ability to achieve a wide range of colors while maintaining relatively high optical transmittance [7]. For example, material combinations with large refractive index contrasts, such as TiN/AlN or MoO_3_/WO_3_, have been reported to enable the fabrication of high-efficiency colored glass, with color variation readily achieved through precise control of thin-film thickness [8]. Nevertheless, this approach still faces challenges related to coating uniformity over large-area substrates and long-term color stability, indicating that further technological improvements are required for commercialization.

One promising technique for overcoming these limitations is radio-frequency (RF) magnetron sputtering, which has recently attracted considerable attention. This physical vapor deposition process involves the ejection of atoms from a target material through ion bombardment generated in a plasma, followed by their deposition onto a substrate. RF magnetron sputtering can be applied to insulating targets and offers precise control over thin-film thickness. Moreover, it enables uniform coating over large-area substrates and allows the use of a wide range of metallic and metal-oxide targets, thereby providing high flexibility in color implementation [9]. When metal-oxide thin films with thicknesses below 100 nm are deposited, various colors can be generated through optical interference effects. In particular, deposition on indium tin oxide (ITO) substrates enables the simultaneous achievement of electrical conductivity and optical transparency, making this approach especially advantageous for BIPV applications. RF sputtering has already been successfully applied in studies of various thin-film photovoltaic technologies, including CdS, CdTe, and Sb_2_Se_3_, demonstrating its effectiveness. In CdS/CdTe thin-film solar cells, for example, optimal optical and electrical properties have been achieved by controlling the CdS layer thickness within the range of 70–135 nm [10]. Similarly, studies on Sb_2_Se_3_-based thin-film solar cells have reported improvements in crystallinity and carrier concentration through the application of RF sputtering [11]. These previous studies suggest that sputtering techniques can be effectively extended beyond conventional photovoltaic thin-film fabrication to the production of colored glass for BIPV applications.

Despite these advances, many existing studies have focused primarily on specific materials or structures, resulting in a lack of comprehensive analysis of the optical and electrical property variations among samples belonging to the same color family. However, for the successful commercialization of BIPV modules, it is essential to consider multiple factors beyond color realization alone, including power generation efficiency, transmittance, reflectance, and durability. The International Energy Agency (IEA) PVPS Task 15 report explicitly states that market acceptance of colored BIPV is closely linked to visual quality, and that architectural requirements such as transparency, color perception, and indoor visual comfort may conflict with the goal of maximizing power generation performance [12]. Nevertheless, many previous studies have relied on qualitative color classification or single metrics such as average transmittance, while systematic analysis of color dispersion ranges and color shift directions within the same color family, based on internationally standardized color spaces, remains limited. Against this background, quantitative colorimetric analysis based on the CIE 1976 L^*^ a^*^ b^*^ (CIELAB) and CIE 1931 chromaticity diagram (CIE 1931) color spaces has emerged as an essential approach for the design and comparative evaluation of colored BIPV glass. In this study, thin films were deposited onto ITO-coated substrates using RF magnetron sputtering to fabricate colored glass samples in the yellow, red, and blue color families. The optical and electrical properties of these samples were comparatively evaluated. In addition, the fabricated specimens were quantitatively assessed using CIELAB coordinates (L^*^, a^*^, b^*^) and CIE 1931 chromaticity coordinates (*x*, *y*), which were presented in tabular form and chromaticity diagrams. Recent studies on colored BIPV glass have primarily focused on achieving specific color tones or improving optical efficiency through material selection and multilayer design. However, systematic analysis of color variation within the same color family, particularly in relation to chromaticity trajectories and material-dependent optical behavior, remains limited. Previous studies have mainly focused on achieving specific colors using single-layer or multilayer coatings or improving optical performance with selected coating materials. In contrast, this study compares different sputtering targets and deposition conditions within the same color family using optical, electrical, and colorimetric analyses, providing a practical basis for material selection in colored BIPV glass. In this context, this study establishes a comparative framework that integrates colorimetric analysis with optical and electrical characterization. The results provide a systematic basis for understanding the relationship between sputtering conditions, material properties, and color realization in BIPV applications. In addition, it is important to note that color generation in thin-film coated glass is fundamentally governed by optical interference effects rather than by sputtering parameters alone. Accordingly, this study discusses the observed color variations based on the measured optical and colorimetric characteristics without attempting to verify the detailed optical mechanism.

## 2. Materials and Methods

**Figure 1:**
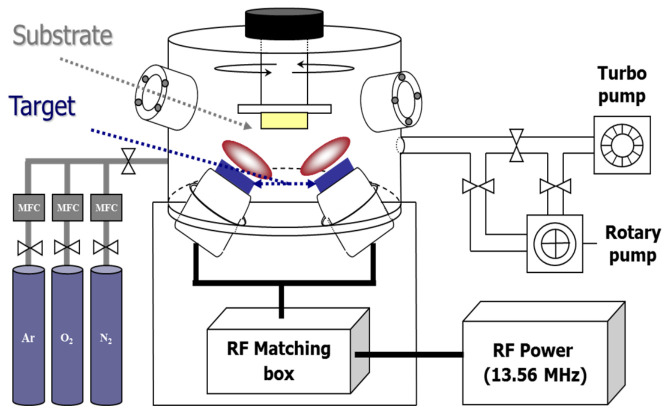
Schematic diagram of the RF magnetron sputtering system.

The experimental procedure consisted of a sequential solvent cleaning process, RF magnetron sputtering deposition, optical characterization, and electrical characterization. To ensure comparability among samples, all experiments were conducted using the same deposition system and consistent substrate preparation procedures. Although absolute control of all process variables was not the primary objective, the relative differences observed among samples can be attributed mainly to the intentionally varied parameters. A schematic illustration of the overall process flow is presented in Figure 1. All deposition experiments were performed under consistent chamber conditions, including base pressure, working gas environment, and substrate configuration, so that the primary variables affecting film properties were limited to the sputtering target, RF power, and deposition time.

**Figure 2:**
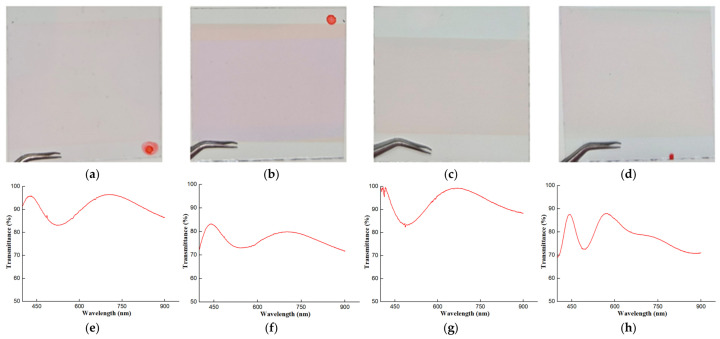
Optical images and Optical transmittance spectra of red-colored glass samples: (**a**,**e**) MoO_3_; (**b**,**f**) MoO_3_/WO_3_; (**c**,**g**) Si; (**d**,**h**) WO_3_.

ITO-coated glass substrates with dimensions of 3.0 × 3.0 cm^2^ were used in the experiments. Prior to deposition, the substrates were cleaned using a sequential solvent cleaning process. Specifically, they were ultrasonically cleaned for 10 min each in acetone, methanol, and deionized (DI) water, followed by drying with nitrogen gas. Thin-film deposition was subsequently carried out using an RF magnetron sputtering system. A schematic diagram of the sputtering apparatus used in this study is shown in Figure 2. All sputtering experiments were conducted under identical deposition conditions. The base pressure was maintained at 1.0 × 10^−5^ Torr, and the working pressure was controlled at 1.5 × 10^−3^ Torr. Argon (Ar) was employed as the sputtering gas at a flow rate of 15 sccm, while the substrate was rotated at 1700 rph during deposition to improve coating uniformity. Unless otherwise stated, these parameters were kept constant for all samples.

This study was intended to compare the optical, electrical, and colorimetric properties of colored glass prepared under different sputtering conditions. Detailed structural characterization was beyond the scope of this work. Ceramic sputtering targets, including metal oxides (MoO_3_, WO_3_, and SiO_2_), a nitride (TiN), and elemental Si, were employed for film deposition. Film thickness was not directly measured in this study using techniques such as ellipsometry or cross-sectional electron microscopy. In addition, detailed microstructural characterization, such as XRD or SEM, was not performed because the focus of this work was placed on the macroscopic optical and electrical responses relevant to BIPV applications. However, under fixed sputtering conditions, deposition time and RF power are generally correlated with film thickness. Accordingly, the variations in sputtering parameters used in this study are expected to produce systematic differences in film thickness. Although multiple factors can influence sputtering outcomes, the present study was designed to investigate the combined effects of material selection and deposition conditions within a consistent experimental framework, rather than to isolate each parameter independently.

## 3. Results

The samples were selected to represent typical coating conditions producing different color tones. Accordingly, the comparisons presented in this study are intended to evaluate the overall optical and colorimetric characteristics of representative colored glass rather than the individual effect of each sputtering parameter. Figure 2 presents optical images of the fabricated colored glass samples exhibiting red color tones after deposition. Figure 2a shows the sample deposited using a MoO_3_ target at an RF sputtering power of 150 W for 15 min. Figure 2b shows the sample sequentially deposited using a MoO_3_ target at 150 W for 15 min, followed by deposition of a WO_3_ layer at 150 W for 30 min. Figure 2c shows the sample deposited using a Si target at an RF sputtering power of 150 W for 30 min. Figure 2d shows the sample deposited using a WO_3_ target at an RF sputtering power of 150 W for 30 min. Before discussing the optical transmittance results, it is necessary to consider the fundamental mechanism governing color formation in thin-film-coated glass. In such systems, color originates from thin-film interference, where partial reflection and transmission occur at interfaces between layers with different refractive indices. The resulting optical path difference between reflected waves leads to wavelength-dependent constructive and destructive interference. In addition to interference effects, the optical response of the deposited films is strongly influenced by intrinsic material properties, including bandgap energy, refractive index, and extinction coefficient. These parameters determine wavelength-dependent absorption and transmission behavior and, consequently, the perceived color of the films. Although the exact film thickness values were not quantitatively measured, the systematic variation observed in optical transmittance and chromaticity suggests that relative differences in optical thickness were consistently achieved across the samples. While numerical approaches such as the Transfer Matrix Method (TMM) can provide quantitative predictions of interference behavior, the present study focuses on experimentally observed optical responses to identify consistent trends across different material systems. Accordingly, the results should be interpreted as trend-based observations rather than as an absolute parameter-dependent optimization [13].

As a result, Color formation in thin-film-coated glass is generally associated with thin-film interference and the optical properties of the coating materials. In this study, these effects are discussed qualitatively based on the measured optical responses.

This study presents a comparative analysis of the optical, electrical, and colorimetric properties of colored glass prepared under different sputtering conditions. Figure 2e through g show the light transmission spectra of colored glass samples belonging to the red series. In addition, from a photovoltaic application perspective, the spectral range of approximately 400–800 nm is particularly important, as it corresponds to the primary photo-response region of silicon-based solar cells. Within this range, the Si-deposited sample exhibits relatively high transmittance compared to other samples, indicating its suitability for BIPV applications where both color realization and power generation efficiency must be considered. The relatively high transmittance observed in the Si-based coating can be attributed to its indirect bandgap structure, which results in lower absorption in the visible range. In contrast, oxide materials such as MoO_3_ and WO_3_ exhibit wavelength-dependent absorption near the band edge, influencing the spectral shape and resulting color characteristics. Figure 2e presents the transmittance spectrum of the sample deposited using a MoO_3_ target at an RF sputtering power of 150 W for 15 min. The measured optical transmittance exhibited an average value of 90.5% in the visible wavelength range from 400 to 800 nm. Figure 2f shows the transmittance spectrum of the sample sequentially deposited using a MoO_3_ target at 150 W for 15 min, followed by a WO_3_ layer deposited at 150 W for 30 min. The average optical transmittance in the 400 to 800 nm range was 75.4%. Figure 2g presents the transmittance spectrum of the sample deposited using a Si target at an RF sputtering power of 150 W for 30 min, yielding an average optical transmittance of 91.2% over the 400 to 800 nm wavelength range. Figure 2h shows the transmittance spectrum of the sample deposited using a WO_3_ target at an RF sputtering power of 150 W for 30 min, with an average optical transmittance of 76.9% in the visible region from 400 to 800 nm.

**Figure 3:**
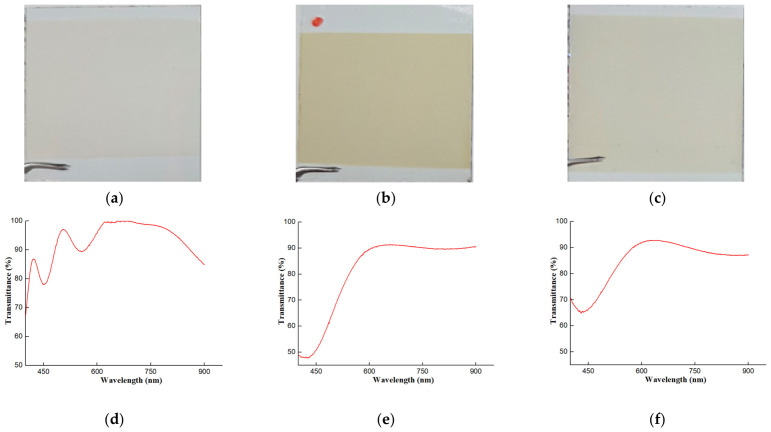
Optical images of yellow-colored glass samples: (**a**,**d**) Si; (**b**,**e**) MoO_3_; (**c**,**f**) TiN.

Figure 3 shows optical images of the fabricated colored glass samples in the yellow color family after deposition. Figure 3a shows the sample deposited using a Si target at an RF sputtering power of 150 W for 60 min. Figure 3b shows the sample deposited using a MoO_3_ target at an RF sputtering power of 50 W for 5 min. Figure 3c shows the sample deposited using a TiN target at an RF sputtering power of 150 W for 35 min.

Figure 3d through f show the optical transmittance spectra of colored glass samples belonging to the yellow color family. Figure 3d presents the transmittance spectrum of the sample deposited using a Si target at an RF sputtering power of 150 W for 60 min. The measured optical transmittance exhibited an average value of 87.6% in the visible wavelength range from 400 to 800 nm. Figure 3e shows the transmittance spectrum of the sample deposited using a MoO_3_ target at an RF sputtering power of 50 W for 5 min. The average optical transmittance in the 400 to 800 nm range was 78.1%. Figure 3f presents the transmittance spectrum of the sample deposited using a TiN target at an RF sputtering power of 150 W for 35 min, yielding an average optical transmittance of 84.3% over the 400 to 800 nm wavelength range. When considering the effective spectral response range of photovoltaic devices (400–800 nm), the Si-based coating maintains relatively high transmittance, whereas the MoO_3_ and TiN coatings exhibit comparatively reduced transmittance in certain wavelength regions. This indicates that material selection significantly influences the balance between optical aesthetics and photovoltaic performance. The shift toward the yellow region observed in oxide-based coatings is associated with increased absorption in the shorter-wavelength (blue) region, which is governed by the material bandgap and electronic transitions. This selective absorption leads to a relative enhancement of longer-wavelength transmission.

**Figure 4:**
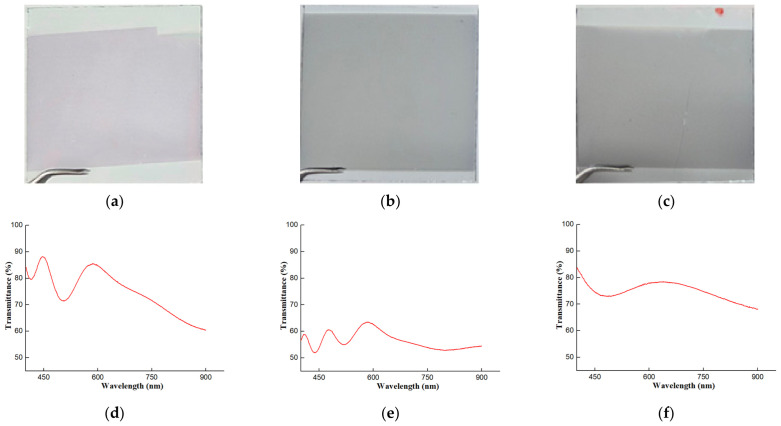
Optical images of blue-colored glass samples: (**a**,**d**) SiO_2_/WO_3_; (**b**,**e**) WO_3_; (**c**,**f**) TiN.

Figure 4 shows optical images of the fabricated colored glass samples in the blue color family after deposition. Figure 4a shows the sample sequentially deposited using a SiO_2_ target at an RF sputtering power of 150 W for 15 min, followed by deposition of a WO_3_ layer at 150 W for 30 min. Figure 4b shows the sample deposited using a WO_3_ target at an RF sputtering power of 200 W for 30 min. Figure 4c shows the sample deposited using a TiN target at an RF sputtering power of 200 W for 30 min.

Figure 4d through f shows the optical transmittance spectra of colored glass samples belonging to the blue color family. Figure 4d presents the transmittance spectrum of the sample sequentially deposited using a SiO_2_ target at an RF sputtering power of 150 W for 15 min, followed by deposition of a WO_3_ layer at 150 W for 30 min. The measured optical transmittance exhibited an average value of 76.3% in the visible wavelength range from 400 to 800 nm. Figure 4e shows the transmittance spectrum of the sample deposited using a WO_3_ target at an RF sputtering power of 200 W for 30 min. The average optical transmittance in the 400 to 800 nm range was 55.6%. Figure 4f presents the transmittance spectrum of the sample deposited using a TiN target at an RF sputtering power of 200 W for 30 min, yielding an average optical transmittance of 75.8% over the 400 to 800 nm wavelength range. In the photovoltaic-relevant spectral range (400–800 nm), the SiO_2_/WO_3_ sample maintains relatively higher transmittance compared to the WO_3_-only sample, suggesting improved compatibility with photovoltaic performance requirements. In contrast, samples with lower transmittance in this range may lead to reduced power generation efficiency despite achieving distinct color characteristics. TiN-based coatings exhibit metallic optical behavior characterized by high free-carrier absorption and reflectance. This results in reduced overall transmittance and a shift toward the blue region in chromaticity space, distinguishing them from oxide-based dielectric coatings.

Table 1 summarizes the average sheet resistance of samples deposited onto ITO-coated glass substrates using RF sputtering with various targets. The sample deposited using a Si target at an RF sputtering power of 150 W for 60 min exhibited the lowest average sheet resistance of 36.2 Ω. In contrast, the sample deposited using a WO_3_ target at 150 W for 30 min showed the highest average sheet resistance of 3.05 MΩ. The measured sheet resistance values reflect the intrinsic electrical properties of the deposited materials. In particular, Si-based coatings exhibited relatively low sheet resistance due to their semiconducting nature, while oxide-based coatings such as WO_3_ showed significantly higher resistance due to their insulating characteristics. TiN-based coatings exhibited intermediate behavior, reflecting their metallic nature. From a BIPV application perspective, the electrical properties of the coating layer influence carrier transport and device integration. Therefore, the observed variation in sheet resistance highlights the trade-off between optical transparency and electrical conductivity in colored glass design.

Table 2 summarizes the colorimetric properties of the colored glass specimens measured under a CIE Standard Illuminant D65 (D65) illuminant, including the CIELAB coordinates (L^*^, a^*^, and b^*^), the CIE 1931 chromaticity coordinates (*x*, *y*), and the color difference (ΔE^*^_ab_) relative to the reference specimen (MoO_3_/WO_3_). The CIE 1931 chromaticity coordinates (*x*, *y*) were calculated based on the CIELAB coordinates (L^*^, a^*^, and b^*^) obtained from the colorimeter measurements.

First, the measured CIELAB coordinates were converted back to the CIE XYZ tristimulus values using the nonlinear transformation defined by the CIE. In this study, the D65 illuminant and the 2° standard observer were adopted, and the corresponding reference white tristimulus values were set to *X*_n_ = 95.047, *Y*_n_ = 100.000, and *Z*_n_ = 108.883 [14]. All colorimetric measurements were performed under the D65 standard illuminant, ensuring consistency with internationally recognized colorimetric standards. The conversion from CIELAB coordinates to *XYZ* tristimulus values was performed as follows:

(1) $$f^{-1}=\left\{\begin{array}{cc} t^{3}\;, & \;t>\frac{6}{29}\\ 3{\left(\frac{6}{29}\right)}^{2}\left(t-\frac{4}{29}\right)\;, & \;t\leq \frac{6}{29} \end{array}\right.$$

(2) $$X=X_{n}f^{-1}\left(\frac{L^{*}+16}{116}+\frac{a^{*}}{500}\right),$$

(3) $$Y=Y_{n}f^{-1}\left(\frac{L^{*}+16}{116}\right),$$

(4) $$Z=Z_{n}f^{-1}\left(\frac{L^{*}+16}{116}-\frac{b^{*}}{200}\right).$$

Using the calculated *XYZ* tristimulus values, the CIE 1931 chromaticity coordinates (*x*, *y*) were obtained by normalization as follows:

(5) $$x=\frac{X}{X+Y+Z},\;y=\frac{Y}{X+Y+Z}.$$

The color difference ΔE^*^_ab_ represents the distance between a reference specimen and a comparison specimen in the three orthogonal coordinate axes of the CIELAB color space, namely lightness (L^*^), the red–green axis (a^*^), and the yellow–blue axis (b^*^). It is a widely used metric that approximately reflects human visual color perception. The color difference in each specimen was calculated using the CIELAB coordinates (L^*^, a^*^, and b^*^) measured by the colorimeter, according to the following equation:

(6) $$∆E_{ab}^{*}=\sqrt{{\left(L^{*}-L_{ref}^{*}\right)}^{2}+{\left(a^{*}-a_{ref}^{*}\right)}^{2}+{\left(b^{*}-b_{ref}^{*}\right)}^{2}}$$

Here, L^*^_ref_, a^*^_ref_, and b^*^_ref_ denote the CIELAB coordinates of the MoO_3_/WO_3_-coated glass selected as the reference specimen, while L^*^, a^*^, and b^*^ represent the measured values of the comparison specimen. The above equation calculates the Euclidean distance between two color coordinates in the CIELAB color space, thereby expressing the overall color difference—combining both lightness and chromaticity differences—as a single scalar value.

While the CIE 1931 color space was used to describe the absolute chromaticity positions of the specimens, color differences were quantified using the CIELAB-based ΔE^*^_ab_ metric following the standardized procedure defined in ISO/CIE 11664-4: 2019 [15]. Although more advanced color difference formulas, such as CIE94 and CIEDE2000, have been proposed to improve perceptual uniformity, the ΔE^*^_ab_ (CIE 1976) formulation remains widely used for comparative colorimetric analysis due to its simplicity, transparency, and standardized implementation, particularly in materials and thin-film coating studies [16,17]. Accordingly, the ΔE^*^_ab_ values reported in this work provide a quantitative index for comparing color shifts induced by different coating combinations under the D65 measurement condition.

As shown in Table 3, the lightness (L^*^) of the specimens ranges approximately from 57 to 92, indicating substantial brightness variation depending on deposition composition and sputtering conditions. In addition, as the b^*^ value increases (shift toward the yellow direction), both *x* and *y* coordinates tend to increase, whereas specimens with negative b^*^ values (blue color family) are located in a relatively lower *x*–*y* region. The color families were classified based on the measured CIELAB coordinates together with visual observation. Samples with positive b values were classified as the yellow color family, whereas samples with negative b values were classified as the blue color family. Samples with relatively high positive a^*^ values were classified as the red color family.

**Figure 5:**
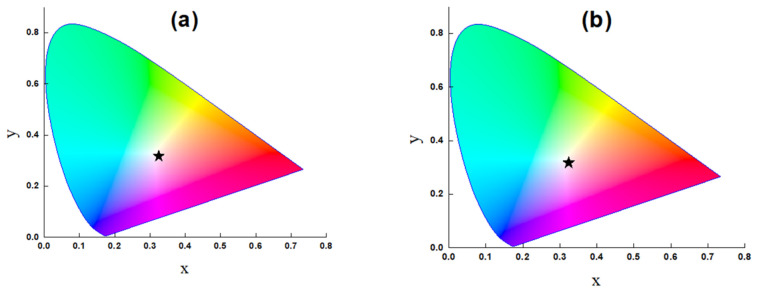

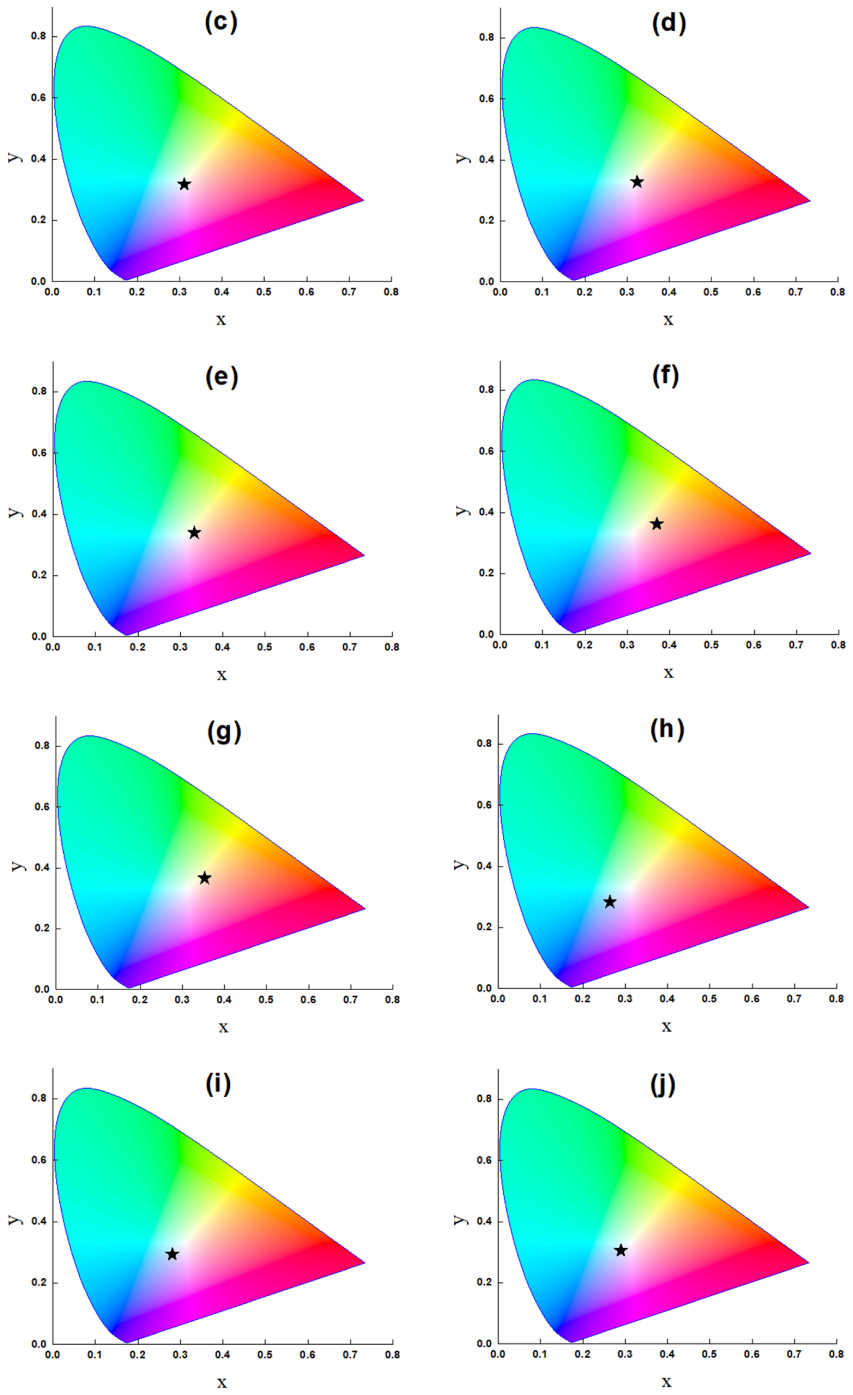
CIE 1931 chromaticity diagrams for individual specimens: (**a**) MoO_3_ + WO_3_ 150 W; (**b**) Si 150 W 30 min; (**c**) MoO_3_ 150 W 15 min; (**d**) WO_3_ 150 W 30 min; (**e**) Si 150 W 60 min; (**f**) MoO_3_ 50 W 5 min; (**g**) TiN 150 W 35 min; (**h**) SiO_2_ + WO_3_ 150 W; (**i**) WO_3_ 200 W 30 min; (**j**) TiN 200 W 30 min.

Figure 5 individually visualizes the CIE 1931 chromaticity diagram for each colored glass specimen to clearly present its absolute chromaticity location. Each diagram is a conceptual illustration in the standard CIE 1931 color space, including the spectral locus and the D65 reference white point, where the chromaticity coordinate of each specimen is marked with a black asterisk. As observed in the individual diagrams, the specimens are distributed at different directions and distances with respect to the D65 white point, reflecting differences in their hue components. Specimens located relatively close to the white point exhibit near-neutral color characteristics, whereas specimens farther from the white point show stronger coloration with enhanced chromatic components. Such absolute coordinate-based chromaticity information provides a basis for interpreting color-shift directions and color-family distribution trends in subsequent comprehensive chromaticity analysis.

In this study, the MoO_3_/WO_3_-deposited specimen was selected as the reference specimen for the calculation of color difference (ΔE^*^_ab_). This choice was motivated by the observation that the MoO_3_/WO_3_ specimen exhibited an intermediate level of lightness (L^*^) and relatively small a^*^ and b^*^ values among the various coating combinations investigated. In addition, its chromaticity coordinate was located near the D65 white point in the CIE 1931 chromaticity diagram. These characteristics indicate that the MoO_3_/WO_3_ specimen can function as a neutral-like reference state that is not excessively biased toward a specific color family. Furthermore, the MoO_3_/WO_3_ coating represents an oxide-based composite structure and exhibits intermediate optical response characteristics compared with the single-oxide coatings (MoO_3_ and WO_3_) and the metal nitride (TiN) coatings included in this study. By adopting the MoO_3_/WO_3_ specimen as the reference, color shifts induced by alternative coating materials and variations in sputtering conditions can be compared in a relative manner without undue bias toward a specific material system. For these reasons, the MoO_3_/WO_3_ specimen is considered a more appropriate internal reference for the colorimetric analysis in this study than industrial colorless glass or a specific single-material coating. It should be noted that, in industrial and standardized colorimetric evaluations, reference states such as uncoated glass or standard illuminants (e.g., D65) are commonly used. However, the primary objective of this study is to compare relative color variations among coated samples within the same experimental framework. Therefore, the MoO_3_/WO_3_-coated specimen was selected as an internal reference to provide a consistent baseline for comparative analysis across different material systems. Accordingly, the ΔE^*^_ab_ values reported in this work provide not only a measure of absolute color difference but also a consistent metric for interpreting the relative magnitude of color changes within the coating design space spanning oxide, composite, and nitride systems.

By jointly analyzing the quantitative colorimetric indicators summarized in Table 3 and the chromaticity-space distributions presented in Figure 5, the color shifts in the colored glass specimens can be interpreted as a multidimensional phenomenon resulting from the combined effects of lightness variation and chromaticity change. This approach enables a systematic comparison of color realization characteristics as a function of coating materials and process conditions.

In this study, to provide more precise selection criteria for BIPV design, the optical and electrical properties of colored glass samples in the yellow, red, and blue color families were comparatively analyzed. The samples were fabricated by depositing thin films onto ITO-coated substrates using RF magnetron sputtering with various targets, including MoO_3_, WO_3_, Si, TiN, and SiO_2_. Among the red-colored glass samples, the specimen deposited using a Si target at an RF sputtering power of 150 W for 30 min exhibited the highest average optical transmittance of 91.2% in the visible wavelength range from 400 to 800 nm, with an average sheet resistance of 5.36 kΩ. For the yellow color family, the highest average optical transmittance of 87.6% (400–800 nm) was obtained for the sample deposited using a Si target at 150 W for 60 min, which also showed the lowest average sheet resistance of 36.2 Ω. In the blue color family, the sample sequentially deposited using a SiO_2_ target at 150 W for 15 min followed by a WO_3_ layer deposited at 150 W for 30 min exhibited the highest average optical transmittance of 76.3% in the 400 to 800 nm range, with an average sheet resistance of 2.88 MΩ. Overall, the experimental results indicate that the use of a Si target leads to the highest optical transmittance across all three color families—red, yellow, and blue—under the investigated sputtering conditions. These findings suggest that Si-based coatings are particularly effective in achieving high optical transparency while maintaining tunable electrical properties, thereby providing useful guidance for material selection in the design of colored glass for BIPV applications.

**Figure 6:**
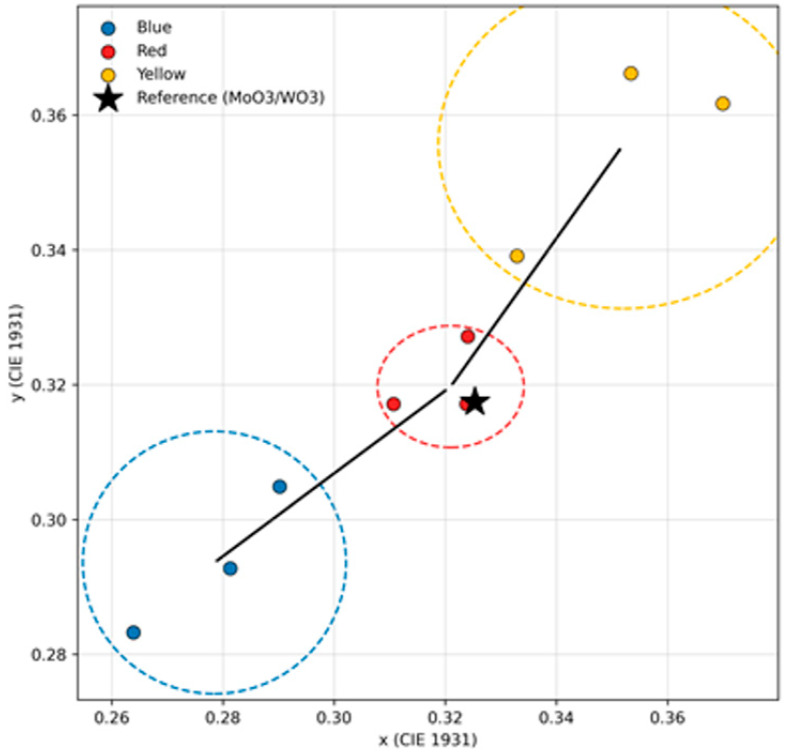
CIE 1931 chromaticity distribution of colored glass specimens measured under the D65 illuminant.

These trends are more clearly illustrated in the CIE 1931 chromaticity diagram presented in Figure 6. Based on the chromaticity coordinates summarized in Table 3, Figure 6 visualizes the color distribution of each specimen within the CIE 1931 chromaticity space. Although all specimens are distributed around the D65 white point, they do not appear randomly scattered; instead, they form continuous trajectories within the chromaticity space. In particular, specimens belonging to the yellow color family with b^*^ > 0 are clustered in the upper-right region of the chromaticity diagram, whereas blue color family specimens with b^*^ < 0, including those deposited using TiN-based coatings, are located in the lower-left region. This distribution indicates that color shifts induced by variations in coating materials and sputtering conditions follow a physically consistent pattern within the color space. These results indicate that the color changes follow systematic trends with sputtering conditions. However, the relationship is discussed qualitatively because film thickness and optical constants were not directly measured. As the film thickness increases, the interference condition shifts across the visible wavelength range, leading to continuous chromaticity trajectories instead of discrete color changes.

Therefore, the correlation between sputtering conditions and color should be interpreted as an indirect relationship mediated by thin-film optical properties. The color difference ΔE^*^_ab_ relative to the reference specimen ranges from approximately 2.379 to 28.226, suggesting that perceptible color differences exist even among specimens with similar lightness levels. These results quantitatively demonstrate that changes in sputtering targets and applied power conditions induce not merely subtle hue adjustments but color variations that are clearly distinguishable by human visual perception. Although explicit statistical error analysis was not included, the consistent trends observed across multiple samples indicate good reproducibility of the experimental results. The systematic variation in chromaticity and transmittance suggests that the observed behavior is governed by controlled changes in deposition parameters rather than random variation. Although theoretical modeling using methods such as TMM could provide further quantitative validation, the continuous and systematic chromaticity trends observed in this study indicate that the color variations are governed by physically consistent optical behavior. Therefore, the experimental results provide a reliable basis for comparative analysis and practical material selection.

In addition, the chromaticity analysis results presented in Table 3 and Figure 6 indicate that the color shifts in the colored glass specimens are not random variations but are systematically induced by the optical properties of the coating materials and changes in the sputtering process conditions. In particular, the continuous migration of chromaticity coordinates observed in the CIE 1931 chromaticity diagram can be interpreted as the cumulative manifestation of wavelength-selective absorption and reflection characteristics of each coating material across the visible spectrum. For MoO_3_- and WO_3_-based depositions, relatively high L^*^ values were maintained while the b^*^ values gradually increased. This trend is attributed to enhanced absorption in the short-wavelength (blue) region accompanied by dominant transmission or reflection in the longer-wavelength (yellow–red) region. Such behavior is consistent with the general optical response of oxide thin films, in which variations in film thickness and composition lead to shifts in the absorption edge within the visible range. In contrast, TiN-based deposited specimens exhibited both low L^*^ values and negative b^*^ values, resulting in a shift toward the lower-left region of the CIE chromaticity diagram. This behavior can be explained by the metallic optical response of TiN thin films, where increased reflectance in the short-wavelength region and overall visible-light absorption occur simultaneously. These characteristics produce a color-shift trend that is clearly distinguishable from that of oxide-based coatings, highlighting the material-dependent differentiation in color implementation strategies. Furthermore, the continuous movement of chromaticity coordinates observed within the same material family as a function of sputtering power indicates that changes in thin-film thickness and microstructure are linked to interference effects and variations in the effective refractive index. This finding suggests that color variation arises not from selective absorption at a single wavelength but from a complex optical response spanning the entire visible region. Accordingly, both material selection and process condition control are identified as key parameters for systematically tuning color realization in colored glass design. The consistent and continuous distribution patterns observed across different samples indicate that the experimental results are not governed by random variations but reflect systematic optical responses under controlled comparative conditions.

Future studies employing a broader range of targets beyond those investigated in this work, in combination with RF magnetron sputtering, are expected to clarify how material properties vary within the same color family. Further studies incorporating detailed thickness measurements and microstructural characterization are expected to provide deeper insight into the relationship between film structure and optical performance. Such investigations would provide more refined selection criteria for BIPV module design and contribute to the development of next-generation BIPV modules that simultaneously satisfy architectural aesthetics and energy efficiency requirements.

## 4. Conclusions

In this study, colored glass for BIPV applications was fabricated using RF magnetron sputtering with various ceramic targets. The optical, electrical, and colorimetric properties of the samples were systematically analyzed across red, yellow, and blue color families. The observed color variations were consistent with thin-film interference and the optical properties of the coating materials. The observed chromaticity distributions exhibit continuous trajectories in CIE color space, indicating physically consistent trends rather than random variation. From an application perspective, analysis within the photovoltaic spectral range (400–800 nm) highlights the importance of material selection in balancing aesthetic design and energy performance. In particular, Si-based coatings exhibit relatively high transmittance, indicating their potential for BIPV applications requiring both transparency and functionality. This study provides a systematic framework for understanding color realization in sputtered thin films and offers practical guidance for material selection and process design in colored BIPV glass. Future work will include direct thickness characterization and optical modeling to further enhance predictive capability. Future work will include film thickness measurements and structural characterization to further clarify the optical mechanism.

## Figures and Tables

**Table 1:** Sheet resistance measurement results of the samples.

Sample Type	Average Sheet Resistance (Ω)
MoO_3_ (50 W, 5 min)	81
MoO_3_ (150 W, 15 min)	1.57 k
MoO_3_ + WO_3_ (150 W)	0.33 M
Si (150 W, 30 min)	5.36 k
WO_3_ (150 W, 30 min)	3.05 M
Si (150 W, 60 min)	36.2
TiN (150 W, 35 min)	1.32 M
SiO_2_ + WO_3_	2.88 M
WO_3_ (200 W, 30 min)	2.23 M
TiN (200 W, 30 min)	39.5 k

**Table 2:** Components of the ΔE^*^_ab_ calculation.

Symbol	Description	Symbol	Description
*L* ^*^	Lightness coordinate	*L* ^*^ *_ref_*	Reference lightness
*a* ^*^	Red–green axis	*a* ^*^ *_ref_*	a^*^ value of reference specimen
*b* ^*^	Yellow–blue axis	*b* ^*^ *_ref_*	b^*^ value of reference specimen

**Table 3:** Colorimetric coordinates (CIELAB and CIE 1931) and color differences in colored glass specimens measured under the D65 illuminant.

Sample	*L* ^*^	*a* ^*^	*b* ^*^	*x*	*y*	ΔE^*^_ab_
MoO_3_ + WO_3_ 150 W	77.107	10.203	−1.768	0.325	0.317	0
Si 150 W 30 min	74.864	9.463	−2.053	0.324	0.317	2.379
MoO_3_ 150 W 15 min	85.185	4.428	−4.407	0.311	0.317	10.275
WO_3_ 150 W 30 min	85.027	6.024	1.222	0.324	0.327	9.441
Si 150 W 60 min	84.072	4.668	6.685	0.333	0.339	12.272
MoO_3_ 50 W 5 min	80.211	10.248	19.913	0.37	0.362	21.902
TiN 150 W 35 min	92.326	2.38	20.68	0.353	0.366	28.226
SiO_2_ + WO_3_ 150 W	57.482	−2.107	−17.309	0.264	0.283	27.896
WO_3_ 200 W 30 min	65.82	1.286	−14.312	0.281	0.293	19.086
TiN 200 W 30 min	64.409	0.17	−9.493	0.29	0.305	17.933

## Data Availability

The original contributions presented in this study are included in the article. Further inquiries can be directed to the corresponding author.
